# A case of malignant hyperlactaemic acidosis appearing upon treatment with the mono-carboxylase transporter 1 inhibitor AZD3965

**DOI:** 10.1038/s41416-020-0727-8

**Published:** 2020-02-20

**Authors:** Rosie McNeillis, Alastair Greystoke, Jon Walton, Chris Bacon, Hector Keun, Alexandros Siskos, George Petrides, Nicola Leech, Fiona Jenkinson, Ann Bowron, Sarah Halford, Ruth Plummer

**Affiliations:** 1 Newcastle upon Tyne Hospitals NHS Foundation Trust, Newcastle upon Tyne, UK; 2 Newcastle University, Newcastle upon Tyne, UK; 3 Imperial College London, London, UK; 4 Centre for Drug Development, Cancer Research UK, London, UK

**Keywords:** Cancer metabolism, Drug development

## Abstract

A 47-year-old man with metastatic melanoma presented with refractory hyperlactaemic acidosis following the first dose of the mono-carboxylase transporter 1 inhibitor AZD3965 within a “first time in man” clinical trial. The mechanism of the agent and the temporal relationship suggested that this event was potentially drug related and recruitment was suspended. However, urinary metabolomics showed extensive abnormalities even prior to drug administration, leading to investigations for an underlying metabolic disorder. The lack of clinical symptoms from the elevated lactate and low blood glucose suggested a diagnosis of “hyper-Warburgism”, where the high tumour burden was associated with extensive glucose uptake and lactate efflux from malignant cells, and the subsequent impact on blood biochemistry. This was supported by an FDG-PET scan showing extensive glucose uptake in numerous metastases and lack of uptake in the brain. A review of the literature showed 16 case reports of “hyper-Warburgism” in non-haematological malignancies, none of them with melanoma, with most associated with a poor outcome. The patient was treated symptomatically, but died 2 months later. The development of AZD3965 continues with the exclusion of patients with elevated plasma lactate at screening added to the protocol as a safety measure.

**Trial identification number** ClinicalTrials.Gov. NCT01791595.

## Background

We present the case of a patient with metastatic melanoma who presented with malignant lactic acidosis, this biochemical abnormality being made clinically apparent by treatment with a novel antitumour agent-targeting metabolism, AZD3965.

Malignant lactic acidosis is a rare complication of solid malignancies, being more common in haematological cancers. An increase in lactate production as a result of the Warburg effect in cancer cells is considered to play an important role in its pathogenesis.^1–4^ The Warburg effect is considered one of the hallmarks of cancer,^5^ and describes the tendency of malignant cells to favour glucose metabolism via glycolysis over oxidative phosphorylation, even in the presence of oxygen. Whilst this appears significantly less efficient for energy production, yielding only two molecules of adenosine triphosphate (ATP) per glucose molecule, compared with 36 produced within oxidative phosphorylation, the process may confer benefit to cancer cells through enhanced availability of metabolic substrates that are essential for cell proliferation, e.g., lipids, amino acids and nucleotides.^2,4,6,7^ Through this metabolic reprogramming, driven by oncogenic genes, such as mTOR, c-MYC and hypoxia-inducible factor 1 (HIF-1), the cancer cells are able to maintain their high proliferation rate.^2,3,6^ Tumour cells can upregulate lactate transporters, in particular the monocarboxylate transporters (MCT) 1 and MCT-4 to remove the excess lactate produced. AZD3965 is an inhibitor of MCT-1 currently in Phase 1/2 clinical development (ClinicalTrials.Gov. NCT01791595) and will inhibit the transport of lactate out of or into cells that do not express MCT-4, the alternative route of excretion. Therefore, it can potentially exploit the dependency of cancer cells on aerobic glycolysis, leading to an accumulation of intracellular lactate, feedback inhibition of glycolysis and pH imbalance.

In this case, a patient with metastatic melanoma presented with severe hyperlactaemic acidosis, following a single dose of the MCT-1 inhibitor, AZD3965, within a clinical trial. Given the temporal relationship with the agent, it was important to determine the cause of this syndrome, both to guide the patient’s immediate management, and also to ensure that further development of this agent could be performed safely.

## Methods

A 47-year-old man with no significant past medical history was diagnosed with BRAF wild-type metastatic melanoma from an unknown primary following needle biopsy of enlarged inguinal lymph nodes. Over the following 18 months, his treatments included inguinal dissection with adjuvant radiotherapy, combination immunotherapy on diagnosis of metastatic disease with ipilimumab and nivolumab, discontinued after three cycles due to grade 4 autoimmune hepatitis, and subsequently three cycles of dacarbazine chemotherapy with progressive disease. He then entered a trial for the first-in-human dose escalation of an oral MCT-1 inhibitor, AZD3965. His baseline trial CT scan demonstrated extensive lymphadenopathy, bone and liver metastases.

Twelve hours after the first dose, he developed severe vomiting unresponsive to anti-emetics. On admission, he was apyrexial but dehydrated with tachycardia (107 bpm) and reduced skin turgor, but normal blood pressure (153/44 mmHg). Arterial blood gas revealed metabolic acidosis (pH: 7.06, standard HCO_3_^−^: 9 mmol/L, base excess: −23 and CO_2_: 2.8 mmHg). Venous lactate was elevated at 7.7 mmol/L (normal range 0.5–2.2 mmol/L). Capillary glucose was 4.1 mmol/L (normal range 4–11 mmol/L). Urine was positive for protein (++), blood (+) and ketones (+++). Imaging revealed no acute changes compared with his baseline scan.

**Fig. 1:**
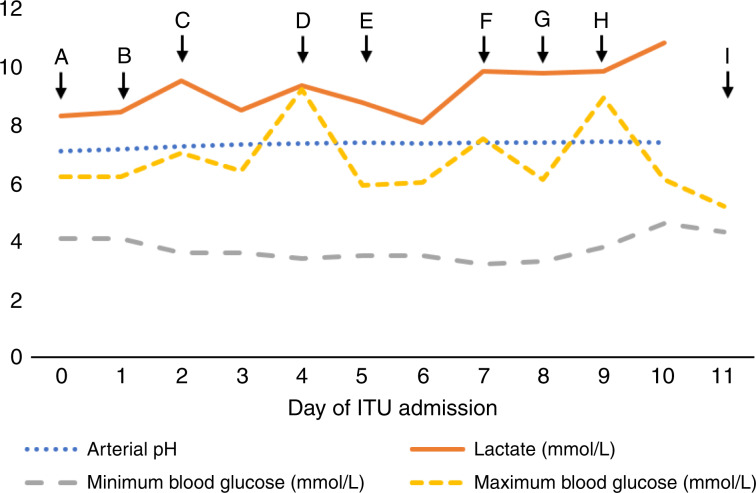
Lactate, arterial pH and blood glucose measured during initial admission to the intensive care unit. Blood glucose was measured on arterial blood gas except on days 9 and 10, where it was recorded as capillary blood glucose. Lactate and pH were not measured on day 11. The highest recorded lactate values and the lowest recorded pH values are shown for each day. A, admission to the ITU. B, started continuous veno-venous haemodiafiltration (CVVHDF). C, started 20% intravenous dextrose. D, stopped intravenous dextrose. E, started intravenous vitamin supplementation (pabrinex). F, stopped CVVHDF. G, started 10% intravenous dextrose. Started oral bicarbonate therapy (2 g of BD). H, oral bicarbonate increased (2 g of TDS). I, transferred to the ward.

Following rehydration, he was transferred to the intensive care unit (ICU) due to persistent hyperlactaemic acidosis (pH: 7.09, lactate 8.29 mmol/L and base excess −21), where he received continuous veno-venous haemodiafiltration (CVVHDF via the Baxter Prismaflex system) and oral sodium bicarbonate (2 g of BD). Throughout admission, he had asymptomatic intermittent hypoglycaemia that was resistant to 20% dextrose infusion, administered from day 2 until day 4, with 10% dextrose infusion given from day 8 onwards, and high calorie intake (see Fig. 1). Serum insulin and C-peptide concentrations were not increased. He remained in the ICU for 10 days for CVVHDF, and IV electrolyte and thiamine supplementation, but despite the severe biochemical abnormalities, he remained clinically well, mobile and ate a normal diet. He was discharged from the ICU to the ward for a further 7 days, and then to home, by taking oral bicarbonate (2 g of TDS) and monitoring his blood sugar via a continuous glucose monitor. Lactate was still elevated at 9.2 mmol/L on discharge.

The seriousness of this event led to suspension of trial recruitment, whilst the study team investigated the cause. No similar toxicity had been observed in any other patient in the trial.

## Results

**Fig. 2:**
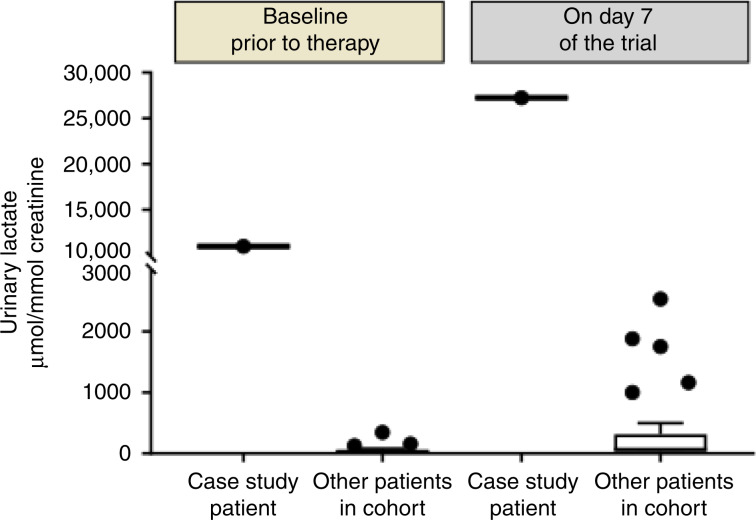
Urinary lactate in the patient before and following treatment with one dose of AZD3965 and stay in the intensive care unit with administration of IV 20% dextrose. Compared with other patients in the clinical trial treated at the same dose of AZD3965. Urinary lactate was measured with ^1^H nuclear magnetic resonance spectroscopy metabolomics analysis.

1H Nuclear Magnetic Resonance spectroscopy analysis revealed high urine ketone and lactate levels in samples taken before the initial dose, with a significant increase following the start of treatment (Fig. 2). The possibility of an underlying inborn error of metabolism unveiled by treatment with AZD3965, such as a disorder of gluconeogenesis or oxidative phosphorylation, was considered. Metabolic investigations were performed, and no abnormalities were identified apart from persistently increased lactic acid in blood and urine (see Table 1). These results, the lack of any previous clinical history suggesting a metabolic disorder and the very mild clinical response to significant acidosis suggested that an inherited metabolic disorder as a cause of lactic acidaemia was unlikely. Given the mechanism of the drug, we also considered congenital MCT-4 deficiency, but strong MCT-4 expression was detected in his original tumour resection samples suggesting that this was unlikely (Supplementary Fig. 1). Pharmacokinetic analysis did not show excessive exposure to AZD3965 compared with previous patients; the maximum concentration of the drug in the patient (*C*_max_) was 71% of the mean values in this cohort, while the estimated exposure over 24 h (AUC_0–24_) was 103% of the mean values in this cohort. The most likely explanation for the persistent lactic acidaemia in this patient was therefore considered to be a result of the abnormal glucose metabolism found in the cancer, and not due to the patient’s underlying physiology.

**Table 1:** The results of key metabolic investigations in the patient.

Investigation	Result	Reference range	Interpretation
Serum insulin (when glucose was 2.2 mmol/L)	<6 pmol/L		Appropriate insulin for hypoglycaemia
Blood lactate	13.4 mmol/L	<1.8	Elevated ratio, not consistent with pyruvate dehydrogenase deficiency
Blood pyruvate	0.28 mmol/L	0.04–0.15	
Blood alanine	1.04 mmol/L	0.2–0.5	
Blood 3-OH butyrate	0.26 mmol/L		Normal ratio
Plasma non-esterified fatty acids	0.65 mmol/L		
Plasma ammonia	33 µmol/L	<50	
Blood spot acylcarnitines	Normal		
Urine organic acids	Elevated lactate and pyruvate, and no increase in Kreb’s cycle intermediates. Elevated ketones with no increase in dicarboxylic acids		Consistent with lactic acidosis and ketosis

The patient was re-admitted 1 week later due to recurrence of hyperlactaemic acidosis (venous lactate 13.8 mmol/L, arterial pH: 7.29), with nausea, upper abdominal discomfort and bloating. His acidosis worsened despite intravenous bicarbonate, and he returned to the ICU for CVVHDF. Symptomatology appeared to be driven by the acidosis rather than lactate or glucose levels; we hypothesised that medical treatment of his hypoglycaemia with glucose (see Fig. 1) was driving increased lactate production in the tumour with the resultant impact on his blood biochemistry; therefore, we started the patient on a ketogenic diet with some improvement in symptoms. A review of the literature at this time found reported cases of “hyper-Warburgism” where in the context of high tumour burden, high glucose uptake in the malignant cells impacts on blood biochemistry, with blood sampling demonstrating low glucose and high lactate as we had observed in this case (see Table 2).

**Table 2:** Reported cases of lactic acidosis in patients with solid malignancies.

Malignancy	Age	Lactate range (mmol/L)	Arterial pH	Liver metastases present	Intervention	Outcome	Ref.
Breast adenocarcinoma	86	7.5–12	7.35	Yes	Thiamine Sodium bicarbonate Chemotherapy	Died (weeks)	^3^
Breast adenocarcinoma	31	16	NS	Yes	Thiamine Sodium bicarbonate Supportive care	Died (8 h)	^19^
Colorectal adenocarcinoma	64	7.2–20.1	6.99	Yes	Sodium bicarbonate Multivitamins Supportive care	Died (6 days)	^11^
Colorectal adenocarcinoma	44	>11	7.24	Yes	Sodium bicarbonate Chemotherapy Starch loading Thiamine Hydrochlor-thiazide	Resolved	^20^
Prostate adenocarcinoma	81	9.5–13.6	7.23	Yes	Chemotherapy Prednisolone Thiamine Sodium bicarbonate	Died (days)	^6^
Gastric adenocarcinoma	81	4.0–6.6	7.43	Yes	Supportive care	Died (days)	^21^
Squamous cell lung cancer	84	13.5–14	7.13	No	Sodium bicarbonate	Died (15 days)	^22^
SCLC	55	26	7.17	Yes	Radiotherapy Chemotherapy	Died (5 days)	^23^
SCLC	57	25.5	7.18	Yes	NS	Died	^23^
SCLC	79	4.5	7.33	Yes	NS	NS	^24^
SCLC	70	15	7.29	No	Chemotherapy Sodium bicarbonate	Resolved	^25^
SCLC	73	4.9–25	6.8	Yes	Sodium bicarbonate Supportive care	Died (days)	^26^
Small-cell carcinoma of the liver	77	13	7.14	Primary in the liver	Supportive care	Died (days)	^27^
CUP	25	171.5	7.08	Yes	Haemodialysis Sodium bicarbonate	Died (8 days)	^28^
CUP	76	7.7	NS	Yes	Sodium bicarbonate CRRT	Died (15 days)	^29^
CUP	14	NS	NS	Yes	Chemotherapy Supportive care	Died (2 months)	^30^
**Melanoma**	**49**	**6.8–16**	**7.05**	**Yes**	**Haemofiltration** **Sodium bicarbonate Multivitamins** **Supportive care**	**Died (2 months)**	

*CRRT* continuous renal replacement therapy, *CUP* carcinoma of unknown primary, *SCLC* small-cell lung cancer.

Previous case reports of lactic acidosis with solid malignancies compared with this case (in bold). Supportive care includes treatments not directly related to relieving acidosis or treating an underlying malignancy, e.g., antibiotics, pantoprazole (for haematemesis), vasopressors, transfusion and standard palliative care. NS: not stated, or for arterial pH only “metabolic acidosis” was stated without giving a value.

**Fig. 3:**
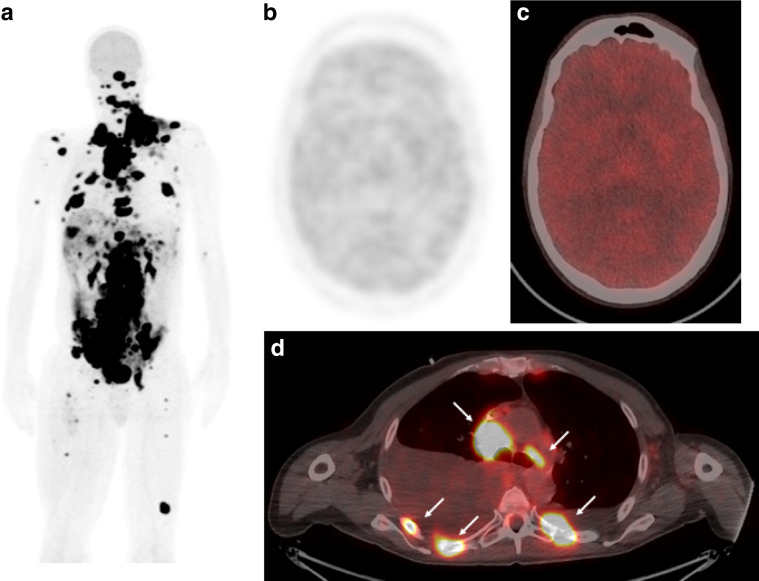
FDG PET showing extensive uptake in tumour metastases throughout the body on maximum intensity projections (a). Reduced uptake in the brain (**b**, **c**) is shown on the maximum intensity projection and fused axial images (hot iron scale) and compared with a cross section of FDG-avid nodal and bone metastases in the thorax (**d**, indicated by white arrows). The PET scan was performed on a GE 710 PET-CT scanner with a dose of 3.5 MBq/kg 18^F^ fludeoxyglucose in 3-min bed positions.

Unfortunately, he was re-admitted 1 month later with recurrent acidosis and a venous lactate of 11.6 mmol/L, which was treated with sodium bicarbonate infusions. A re-staging CT showed disease progression, and a PET scan showed significant volume of FDG-avid disease throughout the body (Fig. 3a, d). We also noted reduced uptake in normal tissues most obvious in the brain, as the organ that has the highest physiological uptake (this is consistent with a change in brain metabolism from glucose to lactate or fatty acid metabolism, Fig. 3b, c). This scan, with his resistant hyperlactaemia and asymptomatic hypoglycaemia, supported a theory of “hyper-Warburgism” as the cause for his hyperlactaemic acidosis. Unfortunately, the patient died 2 weeks later from progressive disease. Baseline lactate-level assessment was added to the protocol as a safety measure for subsequent development of AZD3965.

## Discussion

Lactic acidosis is associated with high mortality,^1,8^ and treatment consists of correcting the underlying cause where possible, and attempting to enhance clearance of lactate and reduce acidosis. Symptomatic treatment with NaHCO_3_ is often used; however, its utilisation is controversial due to lack of an effective response, and a potential association with increased mortality.^8,9^ Other treatments include thiamine supplementation, to encourage oxidation of lactate via the action of pyruvate dehydrogenase,^1^ or haemodialysis to remove excess lactate.^1,10^ In the context of malignancy-related lactic acidosis, case reports suggest that the mainstay of treatment is systemic therapy for the underlying cancer such as chemotherapy; however, the feasibility will depend on the patient’s physiological reserve, cancer type and previous treatment history.^1,11^

In this case, the PET scan showed high glucose uptake by the tumour with extensive disease burden. Melanoma is known to express high levels of GLUT-1,^12^ and in vitro studies show high levels of glycolysis and lactate production.^13–15^ The elevated urinary metabolites prior to therapy, the lack of symptoms with high lactate and low glucose (the patient only became symptomatic when acidotic) and the low glucose uptake in the brain on PET scan all suggest that this was a chronic state. The temporal relationship to treatment and the increase in urinary metabolites following therapy suggest that the AZD3965 precipitated the admission. MCT is responsible for both influx and efflux of lactate, and will reduce import by key tissues such as the liver and kidney. This impact on normal tissues can be most readily observed by the rise in urinary lactate seen in all subjects (Fig. 2). We presume that the single dose temporarily interfered with plasma clearance by the liver and other organs, by precipitating the symptomatic deterioration, and inadvertently the medical team worsened the condition by seeking to reverse the asymptomatic hypoglycaemia. However, in light of the eventual diagnosis and the subsequent continued deterioration, we suspect that this event would have occurred shortly with further disease progression, even in the absence of this treatment.

Case reports of “Hyper-Warburgism” show that in the majority of cases, this was associated with a rapidly fatal outcome (see Table 2); many present themselves unwell with advanced cancer and receive symptomatic measures only. To the authors' knowledge, this is the first report in association with malignant melanoma, with cases more commonly associated with haematological malignancies.^1,3,16–18^ The treatments received in case reports and outcomes are listed in Table 2; only two cases were resolved, both with disease that responded to chemotherapy. Interestingly, a high proportion of cases had primary or metastatic liver involvement (14 out of 16 cases), and the majority of the cases unfortunately resulted in a fatal outcome (13 out of 16). In the presented case, NaHCO_3_ improved the patient symptoms as it helped to correct blood acidosis. However, there has been a link to mortality in cancer-related acidosis,^8^ and NaHCO_3_ can increase acidosis if excess CO_2_ is not cleared effectively. Its use may help protect the heart from the impact of acidosis, until effective systemic therapy can be used, if this is an option.

Overall, this case highlights the potential complexity of clinical development of agents targeting tumour metabolism, the requirement for baseline metabolic assessment before treatment commences and the need for a multidisciplinary team to investigate and manage patients who develop complications on therapy.

## Supplementary information

supplementary figure legend

Supplementary Figure 1

## Data Availability

There are no raw data published in this paper.
