# Correction: A case of malignant hyperlactaemic acidosis appearing upon treatment with the mono-carboxylase transporter 1 inhibitor AZD3965

**DOI:** 10.1038/s41416-020-0801-2

**Published:** 2020-03-12

**Authors:** Rosie McNeillis, Alastair Greystoke, Jon Walton, Chris Bacon, Hector Keun, Alexandros Siskos, George Petrides, Nicola Leech, Fiona Jenkinson, Ann Bowron, Sarah Halford, Ruth Plummer

**Affiliations:** 1 Newcastle upon Tyne Hospitals NHS Foundation Trust, Newcastle upon Tyne, UK; 2 Newcastle University, Newcastle upon Tyne, UK; 3 Imperial College London, London, UK; 4 Centre for Drug Development, Cancer Research UK, London, UK

**Keywords:** Cancer metabolism, Drug development

**Correction to**: *British Journal of Cancer* (2020) 10.1038/s41416-020-0727-8, published online 20 February 2020.

Since the publication of this paper the authors noticed an error in the listed authors, where Alexandros Siskos was listed as Alexandros Sitkos. This has now been corrected.

