# Supplementary material for: A case of malignant hyperlactaemic acidosis appearing upon treatment with the mono-carboxylase transporter 1 inhibitor AZD3965
Source: Br J Cancer. 2020 Feb 20;122(8):1141–5. doi: 10.1038/s41416-020-0727-8 (PMC7156442; doi:10.1038/s41416-020-0727-8)
Supplement: Supplementary file 1 — supplementary figure legend [file 41416_2020_727_MOESM1_ESM.docx]

**Supplementary Figure 1**

Immunohistochemical staining of the patient’s pre-treatment melanoma (A, B) and normal tissue (C) for MCT1 (A) and MCT4 (B, C), showing strong expression of MCT1 and MCT4 by the tumour and strong expression of MCT4 by an eccrine gland. Immunohistochemical staining was performed on the Ventana Benchmark automated staining platform using monoclonal antibodies specific for MCT1 and MCT4 raised in rabbits inoculated with peptides CPDQKDTEGGPKEEESPV and CEPEKNGEVVHTPETSV respectively (as previously used (supplementary Reference 1), and provided by Astra-Zeneca (Cambridge, UK),.

Supplementary Reference

Polanski, R. Hodgkinson C.L. , Fusi, A. et al .Activity of the monocarboxylate transporter 1 inhibitor AZD3965 in small cell lung cancer. Clin Cancer Res. 2014 Feb 15;20(4):926-937. doi: 10.1158/1078-0432.CCR-13-2270. Epub 2013 Nov 25.
